# Supplementary material for: Difference in prioritization of patient safety interventions between experts and patient safety managers in Japan
Source: PLoS One. 2023 Mar 1;18(3):e0280475. doi: 10.1371/journal.pone.0280475 (PMC9977033; doi:10.1371/journal.pone.0280475)
Supplement: S1 Table — (PDF) [file pone.0280475.s001.pdf]

S1 Table. Questionnaire items of evaluation on organizational and clinical level interventions for patient safety in the Delphi survey.

Q: Please rate the following items on a scale of 1 to 5 and write your opinion about various interventions related to patient safety at the organization level, and clinical level.

No.	Intervention	Examples of Intervention	Past contribution to patient safety	Current dissemination	For implementation in the future				Comments on each intervention
					Impact	Cost	Urgency	Priority	
	Organization level								
O-1	Clinical governance frameworks and systems for patient safety	Establish a system to monitor and hold hospitals accountable for the implementation<br>and results of patient safety management							
O-2	Clinical incident reporting and management system	Reporting system in the hospital, a system for investigating and analyzing medical accidents<br>and near-misses, and disseminating remedial measures							
O-3	Integrated patient complaint- and incident-reporting	Opinion boxes, receipt of patient/family opinions by phone, e-mail, etc.,<br>patient consultation services, etc.							
O-4	Monitoring and feedback of patient safety indicators	Promote learning and improvement in clinical practice through feedback of patient safety data<br>(infection rates, readmission rates, patient satisfaction, etc.)							
O-5	Patient-engagement initiatives	Improve patient health literacy (e.g., diabetes classes held at hospitals),<br>provide patients with written explanations, and Shared Decision Making, etc.							
O-6	Clinical communication protocols and training	Standardization of patient information transfer methods during patient transfers, ward transfers, hospital transfers,<br>prevention of information transfer errors, training in listening and communication techniques (TeamSTEPPS, SBAR), etc.							
O-7	Digital technology solutions to improve safety	Electronic medical records, ordering systems, diagnostic imaging support systems, prescription content check systems, etc.							
O-8	Human resources interventions	Staffing standards, bed occupancy, work hours, workload, combination of occupations, etc.							
O-9	Building a positive safety culture	Establishment of patient safety guidelines, education and training, teamwork building,<br>measurement of patient safety culture, awarding of patient safety prizes, etc.							
O-10	Infection detection, reporting and surveillance systems	In-hospital reporting, surveillance, and other systems							
O-11	Hand hygiene initiatives	Ensure hand hygiene and compliance							
O-12	Antimicrobial stewardship	Appropriate use of antimicrobial agents to prevent the development of resistant strains of bacteria							
O-13	Blood and blood product management protocols	Blood sampling for transfusion, cross-match test, patient confirmation, disposal after administration,<br>reporting and investigation of adverse reactions, etc.							
O-14	Medical equipment sterilisation protocols	Sterilization of medical instruments to reduce the risk of infection and the occurrence of drug-resistant bacteria, etc.							
	Clinical level								
C-1	Medication management / reconciliation protocols	Periodic review of patient medications by pharmacists and others, detection of duplicate or omitted medications,<br>medication guidance, prescription audits, etc.							
C-2	Transcribing error systems and protocols	Methods to prevent errors in transcribing drug names, dosage and administration, labeling errors, misreading of handwritten text,<br>etc., and methods for listing similar drug names, etc.							
C-3	Smart infusion pumps and drug administration systems	Highly functional infusion pumps and syringe pumps with patient safety features, alarms under certain conditions,<br>dose limits, decision support, patient identification, etc.							
C-4	Aseptic technique protocols and barrier precautions	Implementation of infection prevention measures, use of gloves and gowns, use of disposable instruments,<br>private rooms and group isolation of infected patients, etc.							
C-5	Urinary catheter use and insertion protocols	Prevention of catheter-related urinary tract infections, proper use, insertion, and management of urinary catheters, etc.							
C-6	Central venous catheter insertion protocols	Prevention of infection, ultrasound-guided insertion, etc.							
C-7	Ventilator-associated pneumonia minimisation protocols	Prevention of infection, fisting of bed head side, sedation, oral care using Hibiten,<br>suctioning of upper cuff of tracheal tube, etc.							
C-8	Procedural / surgical checklists	Preoperative and perioperative checklists to prevent adverse events and infections,<br>WHO Surgical Safety Checklist, etc.							
C-9	Operating room integration and display technology	Technology to integrate or summarize information distributed across multiple devices in the operating room,<br>checklists to prevent information from being missed, etc.							
C-10	Peri-operative medication protocols	Evidence-based perioperative medication procedures to prevent cardiovascular events, deep vein thrombosis, infection, etc.,<br>including beta blockers, antimicrobials, and antithrombotic agents							
C-11	Venous thromboembolism (VTE) prevention protocols	Assessment of risk of deep vein thrombosis, antithrombotic medications, other movement of joints,<br>use of elastic stockings, appropriate fluid intake, etc.							
C-12	Clinical care standards	Application of standard treatment methods for low back pain, acute coronary syndrome,<br>fragile proximal femur fracture, stroke, etc. based on clinical practice guidelines, etc.							
C-13	Pressure injury (ulcer) prevention protocols	Risk assessment of pressure ulcers, method and frequency of postural changes,<br>pressure dispersing bedding, nutrition, skin care, etc.							
C-14	Falls prevention initiatives	Risk assessment of falls, installation of sensors, etc.							
C-15	Acute delirium & cognitive impairment management initiatives	Procedures for early detection and management of delirium and cognitive impairment,<br>procedures for assessment, treatment, and suppression							
C-16	Response to clinical deterioration	Procedures for early detection and management of deteriorating patient conditions,<br>monitoring, appropriate staffing, etc.							
C-17	Patient hydration and nutrition standards	Basic nursing standards and procedures to prevent malnutrition and dehydration							
C-18	Patient identification and procedure matching protocols	Procedures to prevent patient and site misidentification,<br>procedures to prevent surgical site misidentification, etc.							
