# Supplementary material for: Difference in prioritization of patient safety interventions between experts and patient safety managers in Japan
Source: PLoS One. 2023 Mar 1;18(3):e0280475. doi: 10.1371/journal.pone.0280475 (PMC9977033; doi:10.1371/journal.pone.0280475)
Supplement: S2 Table — (PDF) [file pone.0280475.s002.pdf]

S2 Table. Questionnaire items of evaluation on organizational and clinical level interventions for patient safety in the nationwide survey of patient safety managers in hospitals.

Q: Below are the major patient safety interventions that have been implemented to date. Please rate the following items for various system-level, organizational-level, and clinical-level interventions related to patient safety.

No.	Intervention	Examples of Intervention	Implementation status at your hospital	Past contribution	For implementation in the future	
					Priority	Obstacles (Specify)
	Organization level					
O-1	Clinical governance frameworks and systems for patient safety	Establish a system to monitor and hold hospitals accountable for the implementation<br>and results of patient safety management	1. Implemented, 2. Partial,<br>3. Not, 4. Don't know	Small Large<br>1・2・3・4・5	Low High<br>1・2・3・4・5	
O-2	Clinical incident reporting and management system	Reporting system in the hospital, a system for investigating and analyzing medical accidents<br>and near-misses, and disseminating remedial measures	1・2・3・4	1・2・3・4・5	1・2・3・4・5	
O-3	Integrated patient complaint- and incident-reporting	Opinion boxes, receipt of patient/family opinions by phone, e-mail, etc.,<br>patient consultation services, etc.	1・2・3・4	1・2・3・4・5	1・2・3・4・5	
O-4	Monitoring and feedback of patient safety indicators	Promote learning and improvement in clinical practice through feedback of patient safety data<br>(infection rates, readmission rates, patient satisfaction, etc.)	1・2・3・4	1・2・3・4・5	1・2・3・4・5	
O-5	Patient-engagement initiatives	Improve patient health literacy (e.g., diabetes classes held at hospitals),<br>provide patients with written explanations, and Shared Decision Making, etc.	1・2・3・4	1・2・3・4・5	1・2・3・4・5	
O-6	Clinical communication protocols and training	Standardization of patient information transfer methods during patient transfers, ward transfers, hospital transfers,<br>prevention of information transfer errors, training in listening and communication techniques (TeamSTEPPS, SBAR), etc.	1・2・3・4	1・2・3・4・5	1・2・3・4・5	
O-7	Digital technology solutions to improve safety	Electronic medical records, ordering systems, diagnostic imaging support systems, prescription content check systems, etc.	1・2・3・4	1・2・3・4・5	1・2・3・4・5	
O-8	Human resources interventions	Staffing standards, bed occupancy, work hours, workload, combination of occupations, etc.	1・2・3・4	1・2・3・4・5	1・2・3・4・5	
O-9	Building a positive safety culture	Establishment of patient safety guidelines, education and training, teamwork building,<br>measurement of patient safety culture, awarding of patient safety prizes, etc.	1・2・3・4	1・2・3・4・5	1・2・3・4・5	
O-10	Infection detection, reporting and surveillance systems	In-hospital reporting, surveillance, and other systems	1・2・3・4	1・2・3・4・5	1・2・3・4・5	
O-11	Hand hygiene initiatives	Ensure hand hygiene and compliance	1・2・3・4	1・2・3・4・5	1・2・3・4・5	
O-12	Antimicrobial stewardship	Appropriate use of antimicrobial agents to prevent the development of resistant strains of bacteria	1・2・3・4	1・2・3・4・5	1・2・3・4・5	
O-13	Blood and blood product management protocols	Blood sampling for transfusion, cross-match test, patient confirmation, disposal after administration,<br>reporting and investigation of adverse reactions, etc.	1・2・3・4	1・2・3・4・5	1・2・3・4・5	
O-14	Medical equipment sterilisation protocols	Sterilization of medical instruments to reduce the risk of infection and the occurrence of drug-resistant bacteria, etc.	1・2・3・4	1・2・3・4・5	1・2・3・4・5	
	Clinical level					
C-1	Medication management / reconciliation protocols	Periodic review of patient medications by pharmacists and others, detection of duplicate or omitted medications,<br>medication guidance, prescription audits, etc.	1・2・3・4	1・2・3・4・5	1・2・3・4・5	
C-2	Transcribing error systems and protocols	Methods to prevent errors in transcribing drug names, dosage and administration, labeling errors, misreading of handwritten text, etc.,<br>and methods for listing similar drug names, etc.	1・2・3・4	1・2・3・4・5	1・2・3・4・5	
C-3	Smart infusion pumps and drug administration systems	Highly functional infusion pumps and syringe pumps with patient safety features, alarms under certain conditions,<br>dose limits, decision support, patient identification, etc.	1・2・3・4	1・2・3・4・5	1・2・3・4・5	
C-4	Aseptic technique protocols and barrier precautions	Implementation of infection prevention measures, use of gloves and gowns, use of disposable instruments,<br>private rooms and group isolation of infected patients, etc.	1・2・3・4	1・2・3・4・5	1・2・3・4・5	
C-5	Urinary catheter use and insertion protocols	Prevention of catheter-related urinary tract infections, proper use, insertion, and management of urinary catheters, etc.	1・2・3・4	1・2・3・4・5	1・2・3・4・5	
C-6	Central venous catheter insertion protocols	Prevention of infection, ultrasound-guided insertion, etc.	1・2・3・4	1・2・3・4・5	1・2・3・4・5	
C-7	Ventilator-associated pneumonia minimisation protocols	Prevention of infection, fisting of bed head side, sedation, oral care using Hibiten,<br>suctioning of upper cuff of tracheal tube, etc.	1・2・3・4	1・2・3・4・5	1・2・3・4・5	
C-8	Procedural / surgical checklists	Preoperative and perioperative checklists to prevent adverse events and infections,<br>WHO Surgical Safety Checklist, etc.	1・2・3・4	1・2・3・4・5	1・2・3・4・5	
C-9	Operating room integration and display technology	Technology to integrate or summarize information distributed across multiple devices in the operating room,<br>checklists to prevent information from being missed, etc.	1・2・3・4	1・2・3・4・5	1・2・3・4・5	
C-10	Peri-operative medication protocols	Evidence-based perioperative medication procedures to prevent cardiovascular events, deep vein thrombosis, infection, etc.,<br>including beta blockers, antimicrobials, and antithrombotic agents	1・2・3・4	1・2・3・4・5	1・2・3・4・5	
C-11	Venous thromboembolism (VTE) prevention protocols	Assessment of risk of deep vein thrombosis, antithrombotic medications, other movement of joints,<br>use of elastic stockings, appropriate fluid intake, etc.	1・2・3・4	1・2・3・4・5	1・2・3・4・5	
C-12	Clinical care standards	Application of standard treatment methods for low back pain, acute coronary syndrome,<br>fragile proximal femur fracture, stroke, etc. based on clinical practice guidelines, etc.	1・2・3・4	1・2・3・4・5	1・2・3・4・5	
C-13	Pressure injury (ulcer) prevention protocols	Risk assessment of pressure ulcers, method and frequency of postural changes,<br>pressure dispersing bedding, nutrition, skin care, etc.	1・2・3・4	1・2・3・4・5	1・2・3・4・5	
C-14	Falls prevention initiatives	Risk assessment of falls, installation of sensors, etc.	1・2・3・4	1・2・3・4・5	1・2・3・4・5	
C-15	Acute delirium & cognitive impairment management initiatives	Procedures for early detection and management of delirium and cognitive impairment,<br>procedures for assessment, treatment, and suppression	1・2・3・4	1・2・3・4・5	1・2・3・4・5	
C-16	Response to clinical deterioration	Procedures for early detection and management of deteriorating patient conditions,<br>monitoring, appropriate staffing, etc.	1・2・3・4	1・2・3・4・5	1・2・3・4・5	
C-17	Patient hydration and nutrition standards	Basic nursing standards and procedures to prevent malnutrition and dehydration	1・2・3・4	1・2・3・4・5	1・2・3・4・5	
C-18	Patient identification and procedure matching protocols	Procedures to prevent patient and site misidentification,<br>procedures to prevent surgical site misidentification, etc.	1・2・3・4	1・2・3・4・5	1・2・3・4・5	
