# Supplementary material for: Difference in prioritization of patient safety interventions between experts and patient safety managers in Japan
Source: PLoS One. 2023 Mar 1;18(3):e0280475. doi: 10.1371/journal.pone.0280475 (PMC9977033; doi:10.1371/journal.pone.0280475)
Supplement: Attachment — Submitted filename: Response to Reviewers.docx [file pone.0280475.s003.docx]

11/09/2022

Dear Jibril Mohammed, BSc, MSc, PhD

Academic Editor, PLOS ONE

We thank you for the careful evaluation of our manuscript and allowing us to revise. We appreciate your valuable comments and we would like to respond as following:

Journal requirements:

1

Please ensure that your manuscript meets PLOS ONE's style requirements, including those for file naming.

The PLOS ONE style templates can be found at

<https://journals.plos.org/plosone/s/file?id=wjVg/PLOSOne_formatting_sample_main_body.pdf>

and 
<https://journals.plos.org/plosone/s/file?id=ba62/PLOSOne_formatting_sample_title_authors_affiliations.pdf>

Answer:

We checked the format samples and made some corrections to meet PLOS ONE's style requirements, including those for file naming.

2

Please provide additional details regarding participant consent.

In the ethics statement in the Methods and online submission information, please ensure that you have specified what type you obtained (for instance, written or verbal, and if verbal, how it was documented and witnessed).

If your study included minors, state whether you obtained consent from parents or guardians.

If the need for consent was waived by the ethics committee, please include this information.

Answer:

Both surveys did not include minors. We added following explanations.

> P. 6, L. 97-98

All participants of both surveys were informed about the research objective and the policy of data confidentiality and anonymity.

3

In your Data Availability statement, you have not specified where the minimal data set underlying the results described in your manuscript can be found.

PLOS defines a study's minimal data set as the underlying data used to reach the conclusions drawn in the manuscript and any additional data required to replicate the reported study findings in their entirety.

All PLOS journals require that the minimal data set be made fully available.

For more information about our data policy, please see

<http://journals.plos.org/plosone/s/data-availability>.

Answer:

We performed a secondary data analysis of two past surveys. Data were provided for secondary analysis but were not permitted to be published. The aggregated results of the data used for analysis are shown in Table 4 in the paper.

Reviewer 1:

Comment 1

Enhance the rationale for the study to further justify 1. Why these groups may differ in priorities, and 2. What the significance of this is.

Answer:

We corrected and added following explanations.

> P. 5, L. 77-87

Practitioners and patient safety managers are likely to provide patient safety interventions based on the needs and resources on their clinical and organizational settings. Patient safety experts might assess the priority interventions from the perspective of a healthcare system and policy. Therefore, the intervention priorities of safety managers might be different from those of experts. Previous studies have reported the priorities and determinants of patient safety interventions at the clinical level [21,22]. However, few studies have shown differences in intervention priorities between experts and safety managers. By clarifying the differences in prioritization and influential factors, we could identify interventions to be promoted through the support of healthcare system and policies.

Also, we corrected the words related to this point.

> P. 7, L. 118

> P. 8, L. 139

> P. 13, L. 204

> P. 22, L. 249

current ~~present~~

Comment 2

Develop theoretically and empirically the concepts under investigation particularly the notion of the three perspectives: past contribution, present dissemination, and future priority.

These need to be defined (including in the abstract) to be meaningful as well as providing more detail on the wording of questions about these perspectives.

Ideally, and touching on previous comment related to rationale, these concepts would be considered in the introduction since there is an implicit assumption in this study that past contribution might affect future priority etc.

I’d suggest exploring these concepts and their relationship to each other theoretically in addition to describing in the methods how they were defined/assessed, e.g., consider past literature to support these ideas, make hypotheses.

Answer:

We prepared both survey forms focusing on items used in this study as supporting information to provide more detail on the wording of questions about these perspectives.

**S1 Table.** **Questionnaire items of evaluation on organizational and clinical level interventions for patient safety in the Delphi survey.**

**S2 Table.** **Questionnaire items of evaluation on organizational and clinical level interventions for patient safety in the nationwide survey of patient safety managers in hospitals.**

And we added following explanations.

> P. 6, L. 96-97

Parts of both questionnaire items used in this study were shown in S1 and S2 Tables.

Additionally, we corrected and added following explanations.

> P. 4-5, L. 63-76

In this study, the questionnaire consisted of 42 interventions based on the OECD report, and 6 perspectives for assessing the importance of interventions in the past (contribution), current (dissemination), and future (impact, cost, urgency, and priority). They reported that the priority of patient safety interventions had a positive relationship with the future impact and a negative relationship with current dissemination. These results suggested that experts gave high priority to interventions that were expected to be effective in the future and low priority to interventions that were already disseminated. It seemed to be important for policymakers and hospital administrators to consider the status of the medical system, the medical policies that had been taken thus far, and the circumstances that were important in setting the priority of patient safety interventions. These OECD report and previous study suggested that future priorities for interventions might be influenced not only by expected future impact, but also by past contribution and current dissemination.

Comment 3

In methods, explain what makes someone an expert, especially given this group also contains safety managers.

Answer:

To clarify the meaning of this matter, we added following explanations.

> P. 6, L. 108-109

In the Delphi survey, the criteria for experts were to be actively involved in academic activities such as academic conferences or writing papers of patient safety.

Comment 4

Also clarify whether the study was positioned differently for these different groups or whether the wording of questions was slightly different e.g., “for your hospital” for patient safety managers, vs. “national priorities” for patient safety experts. I’d suggest including both survey forms as appendices.

Answer:

We prepared both survey forms focusing on items used in this study as supporting information to provide more detail on the wording of questions about these perspectives.

**S1 Table. Questionnaire items of evaluation on organizational and clinical level interventions for patient safety in the Delphi survey.**

**S2 Table. Questionnaire items of evaluation on organizational and clinical level interventions for patient safety in the nationwide survey of patient safety managers in hospitals.**

And we added following explanations.

> P. 6, L. 96-97

Parts of both questionnaire items used in this study were shown in S1 and S2 Tables.

Additionally, we added the explanation of wording of the questionnaires.

> P. 7-8, L. 133-138

Using the same wording as the Delphi survey for experts, respondents were asked to rate 42 interventions on a 5-point scale from two perspectives: past contribution to patient safety (contribution: 1: small to 5: large) and priority for future implementation (priority: 1: low to 5: high). They were also asked whether 14 interventions at the organizational level and 18 at the clinical level (totaling 32 interventions) were implemented in their hospitals with the wording of “at your hospital”.

Comment 5

Use of a Delphi alongside a traditional survey for comparison throws up a number of issues that need to be addressed: 1. What round of the Delphi responses are the authors using in the analysis? 2. If they are using round three, the authors should consider whether they are comparing experts vs. managers – arguably they are comparing a group that has refined their responses in light of controlled feedback toward a consensus, as compared with another group who has responded to a one off survey.

Answer:

We added following explanations.

> P. 8-9, L. 155-160

As data of experts for current dissemination and future priority, we used the results of round three converged through the Delphi process for analyzing the representative perspectives of patient safety experts in Japan. We included the results of round one on past contribution, as they were only asked at round one. The perspectives of safety managers were varied depending on their own circumstances, there was no need to converge them for the analysis.

Comment 6

Although well-written, there are few instances where wording is overly complicated. For example:

1. Saying “high and low priority by experts and safety managers, respectively” is more complicated than saying “high priority by experts, but low priority by safety managers” and this is a recurrent issue. In addition to revising this wording throughout, it might be useful to create a box or table that summarises the differences e.g., low priority by experts/high priority by managers in one column and high priority by experts/low priority by managers in another. Doing so would also allow some trimming in the Discussion, where these Results are currently repeated. I’d suggest also being explicit on cut offs for when something is deemed low vs high priority.

Answer:

To simplify the expression, the expressions regarding the priority in "Results" are modified as follows.

> P. 3, L. 40-41

> P. 14, L. 208

> P. 14, L. 211-212

high priority by experts, but low priority by safety managers

> P. 14, L. 216

> P. 14, L. 220

high priority by safety managers, but low priority by experts

We added two tables and corrected following explanations.

**Table 6. The interventions that were given high priority by safety managers, but low priority by experts**

**Table 7. The interventions that were given high priority by experts, but low priority by safety managers**

> P. 23, L. 259-260

The interventions that were given high priority by safety managers, but low priority by experts were shown in Table 6.

~~The interventions that were given low and high priority by experts and safety managers, respectively, were “Clinical incident reporting and management system” (O-2), “Medical equipment sterilization protocols” (O-14) in the organization level, “Aseptic technique protocols and barrier precautions” (C-4), “Pressure injury (ulcer) prevention protocols” (C-13), and “Falls prevention initiatives” (C-14) in clinical level.~~

> P. 25, L. 287-288

The interventions that were given high priority by experts, but low priority by safety managers were shown in Table 7.

~~The interventions that were given high and low priority by experts and safety managers, respectively, were “Clinical governance frameworks and systems for patient safety” (O-1), “Patient-engagement initiatives” (O-5), and “Clinical communication protocols and training” (O-6) in the organizational level, “Central venous catheter insertion protocols” (C-6), “Procedural / surgical checklists” (C-8), “Peri-operative medication protocols” (C-10), and “Clinical care standards” (C-12) in the clinical level.~~

To clarify the definition of high and low priority, we added following explanations.

> P. 14, L. 207

We defined scores > 0 as 'high' and < 0 as 'low'.

Reviewer 2:

Comment 1

Dissemination and prioritization can be interpreted differently by various respondents. Were those that participated in the Delphi panel provided definitions of dissemination and prioritization?

Answer:

We prepared both survey forms focusing on items used in this study as supporting information to provide more detail on the wording of questions about these perspectives.

**S1 Table. Questionnaire items of evaluation on organizational and clinical level interventions for patient safety in the Delphi survey.**

**S2 Table. Questionnaire items of evaluation on organizational and clinical level interventions for patient safety in the nationwide survey of patient safety managers in hospitals.**

And we added following explanations.

> P. 6, L. 96-97

Parts of both questionnaire items used in this study were shown in S1 and S2 Tables.

Comment 2

In the analysis the authors state that "Except for the 10 system-level interventions not asked about dissemination in the questionnaire survey of safety managers..." Is there a rationale for why these 10 system level interventions were not included?

Answer:

To clarify the meaning of this matter, we corrected following explanations.

> P. 8, L. 143-148

We assessed the mean values of 32 interventions (Table1), consisting of 14 organizational-level interventions and 18 clinical-level interventions in past contribution, current dissemination, and priority for future implementation. The 10 system-level interventions were not included in the questionnaire for safety managers because these interventions involved the entire national healthcare system and could not be implemented in each hospital.

~~Except for the~~ ~~10 system-level interventions not asked about dissemination in the questionnaire survey of safety managers, we assessed the mean values of 32 interventions (Table1), consisting of 14 organizational-level interventions and 18 clinical-level interventions in past contribution, present dissemination, and priority for future implementation.~~

Comment 3

In the discussion, it would be helpful to more clearly articulate what the potential reasons and implications are for differences in prioritization among experts and patient safety managers.

Answer:

We added following explanations.

> P. 226, L. 300-303

This difference seemed to be caused by that expert's assessment might include a healthcare system and policy perspective, while safety manager's assessment might be based on the needs and resources on their clinical and organizational settings.
