# Supplementary material for: Difference in prioritization of patient safety interventions between experts and patient safety managers in Japan
Source: PLoS One. 2023 Mar 1;18(3):e0280475. doi: 10.1371/journal.pone.0280475 (PMC9977033; doi:10.1371/journal.pone.0280475)
Supplement: Attachment — Submitted filename: Response to Reviewers.docx [file pone.0280475.s004.docx]

27/12/2022

Dear Dr. Keiko Nakamura,

Academic Editor, PLOS ONE

We thank you for the careful evaluation of our manuscript and allowing us to revise. We appreciate your valuable comments and we would like to respond as following:

Journal requirements:

Please review your reference list to ensure that it is complete and correct. If you have cited papers that have been retracted, please include the rationale for doing so in the manuscript text, or remove these references and replace them with relevant current references. Any changes to the reference list should be mentioned in the rebuttal letter that accompanies your revised manuscript. If you need to cite a retracted article, indicate the article’s retracted status in the References list and also include a citation and full reference for the retraction notice.

Answer:

We rechecked all our reference lists and determined everything was fine.

Additional Editor Comments 1

Table 6 and Table 7 should not duplicate the results presented in Table 4a and Table 4b.

Combine Tables 6 and 7 and list the code (for example “O-2”) and interventions (for example “Clinical incident reporting and management system) showing “low priority by experts/high priority by managers” in one column (for example left column) and those showing “high priority by experts/low priority by manages” in another column (for example right column). The number of respondents and scores were presented in Table 4a and Table 4b, therefore these are not needed. A potential title of the updated Table 6 is “A summary list of interventions according to the priorities given by experts and safety managers”.

Answer:

Following your suggestion, we combined Table 6 and Table 7 into new Table 6, revised the structure of Table 6, and changed the title of it.

**Table 6. A summary list of interventions according to the priorities given by experts and safety managers**

| High priority by safety managers / Low priority by experts | | High priority by experts / Low priority by safety manages | |
| --- | --- | --- | --- |
| Organizational level | | Organizational level | |
| O-2 | Clinical incident reporting and management system | O-1 | Clinical governance frameworks and systems for patient safety |
| O-9 | Building a positive safety culture | O-5 | Patient-engagement initiatives |
| O-14 | Medical equipment sterilization protocols | O-6 | Clinical communication protocols and training |
| Clinical level | | Clinical level | |
| C-4 | Aseptic technique protocols and barrier precautions | C-6 | Central venous catheter insertion protocols |
| C-13 | Pressure injury (ulcer) prevention protocols | C-8 | Procedural / surgical checklists |
| C-14 | Falls prevention initiatives | C-10 | Peri-operative medication protocols |
|  |  | C-12 | Clinical care standards |

And, we corrected following explanations in the body.

> P. 22, L. 251

The interventions that were given high priority by safety managers, but low priority by experts were shown in left column of Table 6.

> P. 26, L. 277

The interventions that were given high priority by experts, but low priority by safety managers were shown in right column of Table 6~~7~~.

Additional Editor Comments 2

Confusing sentences are still remain.

“experts and safety managers had given high and low priority, respectively” (lines 284-285)

“experts and safety managers had given high and low priority” (line 322)

Clearer expressions are needed.

Answer:

To simplify, we corrected following explanations.

> P. 26, L. 288-289

were given high priority by experts, but low priority by safety managers

~~experts and safety managers had given high and low priority, respectively~~

> P. 29, L. 328-329

were given high priority by experts, but low priority by safety managers

~~experts and safety managers had given high and low priority~~

We have rechecked our entire manuscript and aligned columns in Table 2b.

| **Table 2a. Baseline characteristics (Experts).** |  |  |
| --- | --- | --- |
|  | n | % |
| Experts | 24 |  |
| Domain |  |  |
| Representative of nationwide organization related to patient safety | 2 | 8.3 |
| Hospital administrator | 5 | 20.8 |
| Patient safety manager | 7 | 29.2 |
| Researcher of patient safety | 8 | 33.3 |
| Other | 2 | 8.3 |
| Profession |  |  |
| Doctor | 15 | 62.5 |
| Nurse | 4 | 16.7 |
| Pharmacist | 2 | 8.3 |
| Others | 3 | 12.5 |
| **Table 2b. Baseline characteristics (Safety managers).** |  |  |
|  | n | % |
| Safety managers | 603 |  |
| Acute care hospital |  |  |
| < 100 beds | 68 | 11.3 |
| 100–299 beds | 178 | 29.5 |
| ≥ 300 beds | 225 | 37.3 |
| Chronic care hospital |  |  |
| < 100 beds | 29 | 4.8 |
| ≥ 100 beds | 48 | 8.0 |
| Psychiatric hospital | 46 | 7.6 |
| Other hospitals | 9 | 1.5 |

At the time of submission, the editorial team pointed out the following corrections.

Journal requirements:

Please provide additional details regarding participant consent.

In the Methods section, please ensure that you have specified (1) whether consent was informed and (2) what type you obtained (for instance, written or verbal).

If your study included minors, state whether you obtained consent from parents or guardians.

If the need for consent was waived by the ethics committee, please include this information.

Answer:

We did not obtain informed consent explicitly for participation in both investigations. However, all participants in both surveys were informed of the purposes of the surveys, data confidentiality and anonymity policies in the request letter. Additionally, taking part in the surveys was voluntary, not mandatory.

Both surveys did not include minors.

Based on our study protocols, the ethics committee did not request us to obtain participant consent.

We added following explanations.

> P. 6, L. 97-99

All participants of both surveys were informed about the research objective and the policy of data confidentiality and anonymity. Taking part in both surveys was voluntary, not mandatory. Therefore, we considered responses to surveys as consent to participate in the survey.
